# Supplementary material for: Embryonic origin of adult stem cells required for tissue homeostasis and regeneration
Source: eLife. 2017 Jan 10;6:e21052. doi: 10.7554/eLife.21052 (PMC5293490; doi:10.7554/eLife.21052)
Supplement: Figure 1—source data 9. — Short summaries describing molecular markers for temporary embryonic tissues and definitive organ systems. Written descriptions accompany Figure 1—figure supplement 11 (primitive ectoderm), Figure 1—figure supplement 12 (temporary embryonic pharynx), Figure 1—figure supplement 13 (gut), Figure 1—figure supplement 14 (definitive pharynx), Figure 1—figure supplement 15 (definitive epidermis), Figure 1—figure supplement 16 (nervous system), Figure 1—figure supplement 17 (muscle), Figure 1—figure supplement 18 (protonephridia) and Figure 1—figure supplement 19 (eyes). DOI: http://dx.doi.org/10.7554/eLife.21052.012 [file elife-21052-fig1-data9.docx]

**Figure 1 – Source Data 9: Molecular Fate Mapping Resource**

**1. Temporary Embryonic Tissues**

**Primitive Ectoderm**

Sphere formation begins during S2 when some of the blastomeres exit the cell cycle and commit to primitive ectoderm fate (Hallez, 1887; Ijima, 1884; Le Moigne, 1963; Metschnikoff, 1883). These cells flatten, elaborate numerous processes, and ultimately generate a single cell layer bounding the sphere. The number of primitive ectoderm cells varied, with a mean of 21.5 +/- 2.9 per S3 embryo (**Figure 1 – figure supplement 11B**). This observation is consistent with primitive ectoderm cell number estimations in *Polycelis nigra* embryos (Le Moigne, 1963). Moreover, the primitive ectoderm cell number remained constant during S4 as the embryo ingested yolk and increased in volume (**Figure 1 – figure supplement 11B-C**). Consistent with this finding, cycling cells were not observed in the primitive ectoderm (**Figure 3A-E**). Pan-embryonic cell markers, including *EF1a-like-1 (SMED30004295)*, *EF1a-like-2* *(SMED30012165)*, and *eIF4a-like* *(SMED30003202)* were expressed in primitive ectoderm cells of S2-S3 embryos, (**Figures 2A-B, 5E, Figure 5 – source data 3**). *gelsolin-like* *(SMED30014940)* was expressed in blastomeres during S2, and primitive ectoderm cells and the temporary embryonic pharynx in S3-S4 embryos (**Figure 1 – figure supplement 11D, Figure 5 – source data 3**). *gelsolin-like* mRNA was detected throughout the primitive ectoderm cell layer, with concentrated perinuclear staining. *spondin-1-like* *(SMED30032088)* was frequently detected in blastomeres during S2, and was expressed in primitive ectoderm cells with star-like morphology, as well as parenchymal cells, in S3-S4 embryos (**Figure 1 – figure supplement 11E**). Early embryo enriched (EEE) transcripts were not detected in the primitive ectoderm beyond S4 (**Figure 5 – source data 3**).

**Temporary Embryonic Pharynx**

The temporary embryonic pharynx is an innervated, muscular pump that ingests yolk into the gut cavity (**Figure 1 – figure supplement 12A-B**). It forms during S2 and likely functions during S3 through S5. Temporary embryonic pharynx-specific markers were identified among the S2-S4 enriched transcripts, including *VAL-like* (**Figure 1 – figure supplement 12C-D**), *netrin-like* (**Figure 1 – figure supplement 12C,E**), *MPEG1-like-1* (**Figure 1 – figure supplement 12C,F**)*, MPEG1-like-2* (**Figure 1 – figure supplement 12C,G**)*.* Expression of *foxA1*, a developmental transcription factor required for maintenance and regeneration of the pharynx during adulthood (Adler et al., 2014; Scimone et al., 2014), was detected in the epithelial cells lining the lumen of the temporary embryonic pharynx during S3-S5 (**Figure 1 – figure supplement 14A**, black arrowheads). The temporary embryonic pharynx degenerates during S6, as the definitive pharynx primordium develops beneath it (Martín-Durán and Romero, 2011). Temporary embryonic pharynx markers are no longer detectable by S7 (**Figure 1 – figure supplement 12C-D**).

**Primitive Gut**

Four yolk-laden primitive gut cells associate with the temporary embryonic pharynx (**Figure 1 – figure supplement 13B-D,** black arrows), and can frequently be detected in unstained specimens. Yolk ingested through the temporary embryonic pharynx collects in the central gut cavity.

**2. Definitive Organ Systems**

**Intestine**

Definitive gut development may begin as early as S4, with the production of isolated phagocytic cells that gradually form a continuous, honeycomb-like lattice beneath the embryonic wall (**Figure 1 – figure supplement 13B-D**). Embryonic gut markers, identified amongst the S4-S5 enriched transcripts, were expressed in gut tissue through S6 and were subsequently downregulated as branching morphogenesis proceeded in elongated S7 embryos (**Figure 1 – figure supplement 13A-D**). The progenitor population(s) for embryonic gut cell type(s) are not known. These early intestinal cells may represent a transient population, turning over during S6-S7, or they may persist, changing their expression signature as morphogenesis proceeds.

Expression of gamma (γ) class neoblast transcripts, including *gata456a* and *hnf4,* was detected in the temporary embryonic pharynx during S2, and in scattered cells in the embryonic wall during S5 (**Figure 1 – figure supplement 13E-G, Figure 6B**); a similar expression pattern was reported for *Spol gata456a* (Martín-Durán and Romero, 2011). The anarchic distribution of putative gut progenitors during embryogenesis is reminiscent of the systemic distribution of gut progenitors in the adult parenchyma, as well as the uniform incorporation of neoblast progeny into the adult gut during growth and homeostasis (Forsthoefel et al., 2011). In the adult gut, neoblast progeny incorporated into both new and preexisting gut tissue during regeneration, and morphallaxis of the gut required stem cell activity (Forsthoefel et al., 2011). Branching morphogenesis begins during S6-S7, as gut cells ingress from the anterior dorsoventral margins of the embryo, forming the secondary branches (**Figure 1 – figure supplement 13F-G, I**). As in the adult, branching morphogenesis may proceed locally and may be reliant upon the incorporation of differentiating progeny, and/or remodeling of differentiated gut tissue. Separation of the two posterior gut branches also occurs during S6-S7, concomitant with the development of the definitive pharynx and a medial, muscular septum running from the pharynx pouch to the posterior pole (**Figure 1 – figure supplement 13F-G, 13I**). Many molecular markers of adult gut tissue are expressed during S5 or later (Forsthoefel et al., 2012; Wurtzel et al., 2015), suggesting that gut maturation is a gradual process (**Figure 1 – figure supplement 13H**). Newborn hatchlings are born with yolk-filled intestines, and it may take up to one week to completely digest and clear the yolk from the gut.

**Definitive Pharynx**

*foxA1*, a pioneer transcription factor required for pharynx maintenance and regeneration during adulthood (Adler et al., 2014; Scimone et al., 2014), may similarly be required for construction of the definitive pharynx during embryogenesis. Development of the definitive pharynx, the single opening of the *Smed* digestive tract, commenced during S4-S5 with the onset of *foxA1* expression in parenchymal cells, many of which were located in the oral hemisphere (**Figure 1 – figure supplement 14A**). The distribution of *foxA1+* cells remained concentrated in and around the developing definitive pharynx rudiment during S6-S8, a pattern reminiscent of that observed in *S. polychroa* embryos (Martín-Durán et al., 2010), as well as in intact and regenerating *Smed* asexual adults (Adler et al., 2014; Scimone et al., 2014). The definitive pharynx develops beneath the degenerating temporary embryonic pharynx, and marks the ventral side of the embryos during S6 and thereafter (Martín-Durán and Romero, 2011). *foxA1* upregulation during S5-S8 was statistically significant, albeit the adjusted p-values were above the thresholds set for inclusion in the enriched transcript lists presented in **Figure 1 – source data 5-8**. *meis,* a transcription factor coexpressed in *foxA1+* neoblasts and expressed within the regenerating pharynx (Scimone et al., 2014), was among the S5 enriched transcripts (**Figure 1 – source data 5**); its expression trend was similar to *foxA1* during embryogenesis (**Figure 1 – figure supplement 14B**). Two markers exhibiting pharynx-restricted expression in adults, *laminin* and *npp-1* (Adler et al., 2014)*,* were upregulated during S6-S8, after development of the definitive pharynx rudiment was evident (**Figure 1 – figure supplement 14B**).

**Definitive Epidermis**

Definitive epidermis development begins during S5, with the advent of *p53* expression in *piwi-1+* cells (**Figure 6A, Figure 1 – figure supplement 15A-B, Figure 1 – source data 5**). *p53* and *zfp-1,* transcriptional regulators upregulated during S5, are required for production of epidermal progenitors in adults, and knock-down of either gene results in loss of the epidermal lineage(s) (Pearson and Sánchez Alvarado, 2010; van Wolfswinkel et al., 2014; Wagner et al., 2012). Epidermal lineage progression during embryogenesis likely utilizes the same transition states elucidated during adult homeostasis. Transcripts expressed in post-mitotic epidermal progenitors, including the Category 2 genes *prog-1 (NB.21.11e), prog-2 (NB.32.1g), prog1-1, prog2-2, and prog2-3,* the Category 3 genes *AGAT-1, AGAT-2, AGAT-3, Cyp1A1, egr-5, SMED30000058, SMED30001567, SMED30002242, pmp-10, SMED30008120, SMED30025584, odc-1/DCOR-1, litaf, pmp-6* and the Category 4 gene *zpuf-6* were among the enriched transcripts in S5 embryos (**Figure 1 – source data 5**) (Eisenhoffer et al., 2008; Tu et al., 2015; van Wolfswinkel et al., 2014; Zhu et al., 2015). Consistent with the RNA-sequencing expression trends, the Category 2 and 3 progenitor markers, *prog-1* and *AGAT-1*, respectively, were first detected by WISH during S5, in scattered parenchymal cells (**Figure 1 – figure supplement 15D-E**). Notably, *p53, prog-1, AGAT-1* and *zpuf-6* positive cells were more numerous on the presumptive dorsal side of elongating S6 embryos (**Figure 1 – figure supplement 15B,D,E,G**), and cells appeared to be more densely packed along the anterior dorsoventral margin. This positional bias in epidermal progenitors was transient, with little difference in the number or density of positive cells observed during S7 or later. Markers of late epidermal progenitor population(s) present within the epidermal monolayer, such as *vim-1, vim-2 and vim-3* (Tu et al., 2015; van Wolfswinkel et al., 2014), showed enriched expression during S7 and S8 (**Figure 1 – source data 7-8**). *vim-3* was first detected by WISH during S6, predominantly on the dorsal side of elongating embryos (**Figure 1 – figure supplement 15H**). By S7, an uninterrupted layer of *vim3+* cells blanketed the embryos, with dorsoventral biases in epidermal cell density becoming less apparent as development proceeded. *crocc (rootletin)*, a marker or terminally differentiated, ciliated epithelial cells (Scimone et al., 2011), was detected during S6 and thereafter (**Figure 1 – figure supplement 15I**).

**Nervous System**

WISH developmental time course data for two markers expressed in differentiated neurons, *synaptotagmin* and *PC-2*, suggests that the nervous system is limited to neurons in the temporary embryonic pharynx of S2-S5 embryos (**Figure 1 – figure supplement 16D, 12A**). Validated and presumptive neural progenitor markers showing low to undetectable expression during early embryogenesis are upregulated as organogenesis commences during S5 (**Figure 1 – figure supplement 16A**) (Cowles et al., 2013; Currie and Pearson, 2013; Lapan and Reddien, 2012; März et al., 2013; Monjo and Romero, 2015; Scimone et al., 2014; Wenemoser et al., 2012), and many transcripts exhibiting enriched expression in adult neurons show upregulated expression during and after S5 (**Figure 1 – figure supplement 16C**). During S6 and S7, neural progenitors and their descendants must migrate, interact, and organize themselves into two bilaterally symmetric cephalic ganglia and the attendant ventral nerve cords, commissural and peripheral neurons (**Figure 1 – figure supplement 16D**). Differentiating neurons accumulate in the presumptive anterior region of the embryo, adjacent to the D/V margin, as the cephalic ganglia form (**Figure 1 – figure supplement 16B,D,** cyan arrows). Ventral nerve cord formation is evident during S6, and appears to proceed from anterior to posterior (**Figure 1 – figure supplement 16D,** cyan arrowheads). Gross morphology of the nervous system is comparable to that of adult animals during S7-S8 (**Figure 1 – figure supplement 16B,D**).

**Muscle**

S2-S4 embryos solely contain radial muscle fibers within the temporary embryonic pharynx (**Figure 1 – figure supplement 17C**). Presumptive progenitors for body wall musculature, cells that coexpress *piwi-1* and *myoD*, emerged during S5 (**Figure 1 – figure supplement 17A-B, Figure 6C**). *myoD* upregulation was first noted during S4, and was significantly enriched in S5 and S6 embryos (albeit the adjusted p-values were higher than the threshold set for inclusion in the S5 and S6 enriched transcript lists) (**Figure 1 – figure supplement 17A-B, Figure 1 – source data 5-6**). While a genetic requirement for *myoD* in muscle cell regeneration has not been demonstrated conclusively during adulthood, *myoD* expression within the adult neoblast compartment has been observed previously (Scimone et al., 2014), and *myoD* knock-down impaired regeneration in two independent reports (Cowles et al., 2013; Reddien et al., 2005). *mhc-1*+ cells were first observed in the parenchyma during S5, without noticeable bias in distribution toward either the oral or aboral hemispheres (**Figure 1 – figure supplement 17C**). Expression within the temporary embryonic pharynx remained strong through S6. During S6, fusiform *mhc-1+* cells became more numerous, and were more densely packed beneath the presumptive dorsal surface of elongating embryos. As elongation proceeded, *mhc-1+* cells were densely packed along the dorsoventral margin, particularly in the anterior half of the embryo (**Figure 1 – figure supplement 17C**). The dorsal bias in *mhc-1+* cell density was transient, and was not obvious by S7. *myoD+* cells were located around the developing cephalic ganglia and ventral nerve cords, in S6-S8 embryos (**Figure 1 – figure supplement 17A**). Concentrations of *myoD+* cells, as well as *mhc-1+* muscle cells, were seen around the definitive pharynx rudiment and along the developing tail stripe, a muscular septum that bifurcates the posterior gut and connects the dorsal and ventral body wall musculature (**Figure 1 – figure supplement 17A,C**). Generation of robust, increasingly dense transverse and longitudinal body wall muscle fiber networks and enteric musculature proceeded during S7-S8 (**Figure 1 – figure supplement 17C**).

**Excretory System**

The transcription factors *pou2/3, six1/2-2, eya, sal1*, all of which were required for protonephridia specification during regeneration, as well as *osr*, a transcription factor expressed in protonephridial progenitors, showed enriched expression during S5 (**Figure 1 – figure supplement 18A, Figure 1 – source data 5**) (Scimone et al., 2011). Scattered *pou2/3+* cells were detected by WISH in S5 embryos (**Figure 1 – figure supplement 18B**), and preferential accumulation of *pou2/3+* cells around the presumptive D/V margin of the embryo was observed as tubules began to form during S6. Branching morphogenesis of the tubules proceeded during S6-S8, with a noted dorsolateral bias in positioning of the developing filtration units (**Figure 1 – figure supplement 18D**). Transcripts with enriched expression in adult protonephridial cell type(s) were frequently expressed at low to undetectable levels prior to S5, and were upregulated during S5-S8 (**Figure 1 – figure supplement 18C**) (Scimone et al., 2011; Vu, 2015; Wurtzel et al., 2015). *crocc (rootletin)*, a marker of ciliated epithelial cells (Scimone et al., 2011), was expressed in the developing protonephridia during S6-S8 (**Figure 1 – figure supplement 15I**).

**Eyes**

Genes required for production of all eye progenitors *(eya, six-1/2*), photoreceptor neuron differentiation (*otxA*), or pigment cup differentiation (*sp6-9, dlx*) were enriched during S5-S6 (**Figure 1 – figure supplement 19A, Figure 1 – source data 5-6**)(Lapan and Reddien, 2011, 2012). The transcription factor *ovo*, a master regulator of eye development, showed statistically significant upregulation during S6, albeit with adjusted p-values and fold-changes that precluded its inclusion on the S6 enriched transcript list (Lapan and Reddien, 2012). *ovo* expression was first detected by WISH during S5-S6, in only a few cells per embryo (**Figure 1 – figure supplement 19B**). During S7, two dorsolateral trails of *ovo*+ progenitors appeared along the anterior head margin, and these cells and their differentiating descendants likely generate the eye primordia (**Figure 1 – figure supplement 19B**, blue arrowheads) (Lapan and Reddien, 2012). The photoreceptor neurons, marked by *opsin* (**Figure 1 – figure supplement 19D**), and the non-neuronal pigment cups, marked by *tyrosinase* (**Figure 1 – figure supplement 19E**), were first visible during S7, consistent with published expression patterns for *Spol*-*opsin* and *Spol-tph* in elongated embryos (Martín-Durán et al., 2012). Growth of the eyes continued through S8. Moreover, co-expression of *ovo* and transcription factors required during eye differentiation, including *eya, klf, six-1/2, soxB, sp6-9, otxA, meis* and *FoxQ2,* was reported in elongated embryos (S7-S8) (Lapan and Reddien, 2012), suggesting that eye development during embryogenesis and adulthood may rely upon common genes and genetic regulatory networks.

**References**

Adler, C.E., Seidel, C.W., McKinney, S.A., and Sánchez Alvarado, A. (2014). Selective amputation of the pharynx identifies a FoxA-dependent regeneration program in planaria. Elife 3, e02238.

Cowles, M.W., Brown, D.D., Nisperos, S.V., Stanley, B.N., Pearson, B.J., and Zayas, R.M. (2013). Genome-wide analysis of the bHLH gene family in planarians identifies factors required for adult neurogenesis and neuronal regeneration. Development 140, 4691-4702.

Currie, K.W., and Pearson, B.J. (2013). Transcription factors lhx1/5-1 and pitx are required for the maintenance and regeneration of serotonergic neurons in planarians. Development 140, 3577-3588.

Eisenhoffer, G.T., Kang, H., and Sánchez Alvarado, A. (2008). Molecular analysis of stem cells and their descendants during cell turnover and regeneration in the planarian Schmidtea mediterranea. Cell Stem Cell 3, 327-339.

Forsthoefel, D.J., James, N.P., Escobar, D.J., Stary, J.M., Vieira, A.P., Waters, F.A., and Newmark, P.A. (2012). An RNAi screen reveals intestinal regulators of branching morphogenesis, differentiation, and stem cell proliferation in planarians. Dev Cell 23, 691-704.

Forsthoefel, D.J., Park, A.E., and Newmark, P.A. (2011). Stem cell-based growth, regeneration, and remodeling of the planarian intestine. Dev Biol 356, 445-459.

Hallez, P. (1887). Embryogénie des Dendrocoeles d’eau douce, Vol 16.

Ijima, I. (1884). Untersuchungeniiber den Bau und die Entwicklungsgeschichte der stisswasser-Dendrocoelen(Tricladen). Vol Bd. 40.

Lapan, S.W., and Reddien, P.W. (2011). dlx and sp6-9 Control optic cup regeneration in a prototypic eye. PLoS Genet 7, e1002226.

Lapan, S.W., and Reddien, P.W. (2012). Transcriptome analysis of the planarian eye identifies ovo as a specific regulator of eye regeneration. Cell Rep 2, 294-307.

Le Moigne, A. (1963). Etude du developpement embryonnaire de Polycelis nigra (Turbellarie - Triclade). Bulletin de la Societe Zoologique de France 88, 403-422.

Martín-Durán, J.M., Amaya, E., and Romero, R. (2010). Germ layer specification and axial patterning in the embryonic development of the freshwater planarian Schmidtea polychroa. Dev Biol 340, 145-158.

Martín-Durán, J.M., Monjo, F., and Romero, R. (2012). Morphological and molecular development of the eyes during embryogenesis of the freshwater planarian Schmidtea polychroa. Dev Genes Evol 222, 45-54.

Martín-Durán, J.M., and Romero, R. (2011). Evolutionary implications of morphogenesis and molecular patterning of the blind gut in the planarian Schmidtea polychroa. Dev Biol 352, 164-176.

März, M., Seebeck, F., and Bartscherer, K. (2013). A Pitx transcription factor controls the establishment and maintenance of the serotonergic lineage in planarians. Development 140, 4499-4509.

Metschnikoff, E. (1883). Die embryologie von Planaria polychroa. Zeitschrift fur wissenschaftliche Zoologie 38, 331-354.

Monjo, F., and Romero, R. (2015). Embryonic development of the nervous system in the planarian Schmidtea polychroa. Dev Biol 397, 305-319.

Pearson, B.J., and Sánchez Alvarado, A. (2010). A planarian p53 homolog regulates proliferation and self-renewal in adult stem cell lineages. Development 137, 213-221.

Reddien, P.W., Bermange, A.L., Murfitt, K.J., Jennings, J.R., and Sánchez Alvarado, A. (2005). Identification of genes needed for regeneration, stem cell function, and tissue homeostasis by systematic gene perturbation in planaria. Dev Cell 8, 635-649.

Scimone, M.L., Kravarik, K.M., Lapan, S.W., and Reddien, P.W. (2014). Neoblast specialization in regeneration of the planarian Schmidtea mediterranea. Stem Cell Reports 3, 339-352.

Scimone, M.L., Srivastava, M., Bell, G.W., and Reddien, P.W. (2011). A regulatory program for excretory system regeneration in planarians. Development 138, 4387-4398.

Tu, K.C., Cheng, L.C., Tk Vu, H., Lange, J.J., McKinney, S.A., Seidel, C.W., and Sánchez Alvarado, A. (2015). egr-5 is a post-mitotic regulator of planarian epidermal differentiation. Elife 4, e10501.

van Wolfswinkel, J.C., Wagner, D.E., and Reddien, P.W. (2014). Single-cell analysis reveals functionally distinct classes within the planarian stem cell compartment. Cell Stem Cell 15, 326-339.

Vu, H.T.-K., Jochen C Rink, Sean A McKinney, Melainia McClain, Naharajan Lakshmanaperumal, Richard Alexander, Alejandro Sánchez Alvarado (2015). Stem cells and fluid flow drive cyst formation in an invertebrate excretory organ. Elife 4, e07405.

Wagner, D.E., Ho, J.J., and Reddien, P.W. (2012). Genetic regulators of a pluripotent adult stem cell system in planarians identified by RNAi and clonal analysis. Cell Stem Cell 10, 299-311.

Wenemoser, D., Lapan, S.W., Wilkinson, A.W., Bell, G.W., and Reddien, P.W. (2012). A molecular wound response program associated with regeneration initiation in planarians. Genes Dev 26, 988-1002.

Wurtzel, O., Cote, L.E., Poirier, A., Satija, R., Regev, A., and Reddien, P.W. (2015). A Generic and Cell-Type-Specific Wound Response Precedes Regeneration in Planarians. Dev Cell 35, 632-645.

Zhu, S.J., Hallows, S.E., Currie, K.W., Xu, C., and Pearson, B.J. (2015). A mex3 homolog is required for differentiation during planarian stem cell lineage development. Elife 4, e07025.
